# Big brother: the effects of surveillance on fundamental aspects of social vision

**DOI:** 10.1093/nc/niae039

**Published:** 2024-12-10

**Authors:** Kiley Seymour, Jarrod McNicoll, Roger Koenig-Robert

**Affiliations:** Faculty of Health, University of Technology, Sydney, New South Wales, Australia; The MARCS Institute, Western Sydney University, Westmead, New South Wales, Australia; High-Field Magnetic Resonance, The Max Planck Institute for Biological Cybernetics, Tübingen, Germany; Faculty of Health, University of Technology, Sydney, New South Wales, Australia; Faculty of Health, University of Technology, Sydney, New South Wales, Australia; School of Psychology, The University of New South Wales, Sydney, NSW, Australia

**Keywords:** privacy, surveillance, technology, consciousness, social cognition, being watched, eye gaze

## Abstract

Despite the dramatic rise of surveillance in our societies, only limited research has examined its effects on humans. While most research has focused on voluntary behaviour, no study has examined the effects of surveillance on more fundamental and automatic aspects of human perceptual awareness and cognition. Here, we show that being watched on CCTV markedly impacts a hardwired and involuntary function of human sensory perception—the ability to consciously detect faces. Using the method of continuous flash suppression (CFS), we show that when people are surveilled (*N* = 24), they are quicker than controls (*N* = 30) to detect faces. An independent control experiment (*N* = 42) ruled out an explanation based on demand characteristics and social desirability biases. These findings show that being watched impacts not only consciously controlled behaviours but also unconscious, involuntary visual processing. Our results have implications concerning the impacts of surveillance on basic human cognition as well as public mental health.

**Highlights**

- Rapid technological advancements have resulted in increasingly powerful forms of human surveillance, but the effects on cognition are unknown.
- We show that being conspicuously monitored via CCTV markedly impacts a hardwired and involuntary function of human sensory perception—the ability to consciously detect a face.
- Surveilled participants became aware of face stimuli almost a second faster than the control group.
- This perceptual enhancement occurs outside the individual’s awareness.

## Introduction

In recent years, we have seen an exponential increase in human surveillance. We now live in a world with closed-circuit television (CCTV) in public spaces, trackable mobile devices, and the monitoring of our activities through artificially intelligent technology and the ‘Internet of Things’ (the interconnected system of our devices and sensors collecting and sharing data through the internet). Data on what we do, what we say, and where we go can be monitored and made available to third parties (Zuboff 2015, Cecez-Kecmanovic 2019). With the advent of emerging neurotechnology, even our mental privacy is at risk (Farahany 2023). Despite this proliferation of surveillance technology, there is limited research on its effects on human psychology, including fundamental capacities like the basic perceptual processing of our sensory environment.

Literature available on the topic of human surveillance and being watched suggests that it elicits changes in overt behaviour. For instance, a large body of evidence on ‘audience effects’ suggests people act in a more prosocial manner when they believe they are being watched. When people think their behaviour is monitored, they are more giving (Hoffman et al. 1996, Haley and Fessler 2005, Pfeiffer and Nowak 2006, Rigdon et al. 2009, Powell et al. 2012, Nettle et al. 2013, Bateson et al. 2015), more likely to share (Baillon et al. 2013, Oda et al. 2015), and less likely to steal, cheat, litter, or direct their gaze to provocative images (Tourangeau and Yan 2007, Zhong et al. 2010, Risko and Kingstone 2011, Francey et al. 2012, Nettle et al. 2012, Nasiopoulos et al. 2015). It is argued that these behavioural changes act to uphold the reputation of the individual and protect from negative social consequences (Izuma 2012, Nettle et al. 2013, Conty et al. 2016).

In addition to the changes in social behaviour, a feeling of being watched commonly invokes discomfort in people (Panagopoulos and Van Der Linden 2017) and increases vigilance, self-consciousness, and the fight-or-flight response (e.g. an increase in heart rate and skin conductance) (Kleinke and Pohlen 1971, Nichols and Champness 1971, Gale et al. 1975, Putz 1975, Reddy 2003, Conty et al. 2010b, Helminen et al. 2011, Baltazar et al. 2014). It has also been shown that surveillance in the workplace induces negative effects on productivity (Gagné and Deci 2005), likely due to impacts on attention and working memory (Senju and Hasegawa 2005, Conty et al. 2010a, Risko and Kingstone 2011, Wang and Apperly 2017, Colombatto et al. 2019). Interestingly, it seems to be an implied social presence rather than a true presence of the observer that is important here, with simple photos of watching eyes or a mere belief that someone is watching eliciting the behavioural changes (Putz 1975, Haley and Fessler 2005, Bateson et al. 2006, Burnham and Hare 2007, Rigdon et al. 2009, van Rompay et al. 2009, Risko and Kingstone 2011, Lawson 2015, Nasiopoulos et al. 2015, Colombatto et al. 2019).

While the effects of surveillance on social behaviour are well-documented, it is unclear how being watched impacts more fundamental capacities not subject to explicit, overt, and conscious control of the individual. For instance, being able to rapidly detect when someone or something is looking at you is a profound and hardwired human faculty requiring specialized neural mechanisms that operate largely outside of conscious control (Brothers 1990, Perrett and Emery 1994, Baron-Cohen 1995, Emery 2000, Calder et al. 2007, Hietanen et al. 2008, Senju and Johnson 2009, Bayliss et al. 2011, Burra et al. 2013, Carlin and Calder 2013). In fact, this heightened sensitivity to another’s gaze is thought to underlie a feeling of being watched that can be experienced in the absence of any surveillance and commonly reported in the population (Freeman et al. 2005, Taylor et al. 2009, Bebbington et al. 2013, Harper and Timmons 2021). Given the adaptive significance, we hypothesize that these mechanisms are further engaged when one knows they are being watched. Indeed, evidence from clinical research suggests that patients with schizophrenia who experience persecutory delusions (i.e. erroneous beliefs about being watched) show increased perceptual sensitivity to the self-directed gaze of others (Rosse et al. 1994, Hooker and Park 2005, Tso et al. 2012, Langdon et al. 2017).

In the current study, we test whether being watched influences perceptual processing of the sensory environment, namely the processing of eye gaze. Specifically, we ask whether being monitored makes the visual system more sensitive to this essential visual and social cue. Using a technique known as breaking continuous flash suppression or b-CFS (Tsuchiya and Koch 2005), we temporarily suppressed photographs of faces from visual awareness. The time the face takes to break through the suppressive mask and become visible to the participant is typically treated as an index of its salience. In previous experiments using b-CFS, it has been shown that the visual system prioritizes the detection of faces with direct gaze over faces with averted gaze, suggesting visual cues used to discriminate eye gaze direction are preconsciously processed by the visual system (Stein et al. 2011, Yokoyama et al. 2013, Seymour et al. 2016). In the current study, we examined whether being watched influenced the speed at which these gaze signals reach conscious awareness by means of a detection task (i.e. stimulus on the left or right). We hypothesized that if being surveilled facilitates basic sensory processing of eye gaze, then participants who had evidence of being monitored during the task (i.e. experiencing the presence of CCTV) would detect self-directed gaze signals faster than participants who did not.

## Method

### Participants

**Figure 1:**
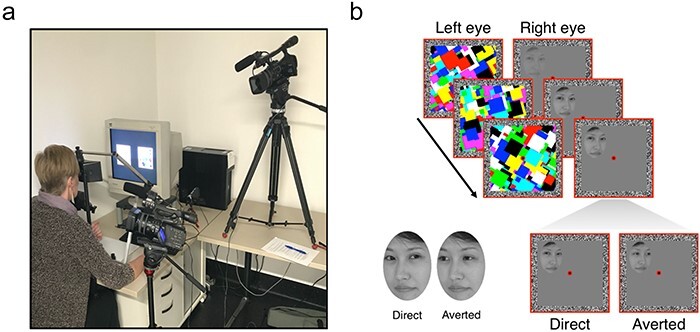
**a**. Experimental setup for the “watched” group. Prior to completing the b-CFS task, participants were shown a live feed of the testing booth from the adjacent room. During the task, four cameras faced the participant. One large camera on a large tripod faced the participant’s whole body from the front, and another was placed in close proximity to their face. A small camera positioned over the keyboard was directed at the participant’s hand during the task. **b**. Schematic of a b-CFS trial. Here, a dynamic mask is presented to the dominant (left) eye, which acts to temporarily suppress the face stimulus presented to the non-dominant (right) eye from reaching conscious awareness. Participants were required to indicate on which side of the central fixation point the face breaks suppression and becomes visible. Faces were presented with either direct gaze or averted gaze

The main study consisted of 54 undergraduate students (5 m/48 F/1 non-binary) who were awarded course credit for their time. Participants had normal or corrected to normal vision and gave written informed consent, which was approved by Western Sydney University’s Human Ethics Committee (ethics approval number H127571). In an attempt to strictly isolate the effects of surveillance, we compared a randomly assigned control group where participants were left alone in the room to complete a detection task (*N* = 24; 6 m/18 F, mean age 21.3) to a randomly assigned experimental group where participants were left alone to complete the same task but watched on closed-circuit television (CCTV) from the adjacent room (*N* = 30; 3 m/26 F/1 non-binary, mean age 21.6). To convince participants in the watched group that they were being surveilled during the task, cameras were noticeably set up within the testing booth prior to testing (Fig. 1a), and participants were shown a live feed of the testing booth from the adjacent room. We chose to make this manipulation conspicuous rather than subtle as it has been shown that the feeling of being watched is already prevalent in healthy participants (Freeman et al. 2005, Taylor et al. 2009, Bebbington et al. 2013, Elahi et al. 2017, Harper and Timmons 2021). These participants were also asked to provide signed consent to being monitored during the experiment. For the control participants, cameras were removed from the testing booth and the live feed monitor was switched off. The allocation of participant groups and associated experimental onboarding was also used in a separate post hoc experiment designed to control for the possibility of demand characteristics influencing the results (i.e. the potential that being watched could cue participants to respond in a way that fulfils the perceived expectations of the experimenter). Here, we ran the same experiment on 42 participants using neutral stimuli (not eye gaze stimuli). Our sample sizes were chosen based on estimated effect sizes from previous studies. With the sample size chosen for the main experiment (*N* = 45), we achieved a power (1 − β) of 0.95 for the group comparison *Watched* vs *Controls* (α = 0.05, two-sample t-test). Similar considerations were used for the separate post hoc experiment (*N* = 42), achieving a power (1 − β) of 0.83 for the group comparison *Watched Neutral Stimuli* vs *Watched Faces* (α = 0.05, two-sample t-test). Post hoc power analyses were performed using GPower3 (Erdfelder et al. 2009).

### Apparatus and stimuli

We closely followed a b-CFS protocol previously used to measure the automatic and unconscious processing of eye gaze direction in human participants (Stein et al. 2011), as shown in Fig. 1b. Stimuli were viewed on a CRT computer monitor through a mirror stereoscope (resolution: 1024 × 768, 60 Hz). Two adjacent red square frames (10.6° × 10.6°) were displayed such that only one frame was visible to each eye. We confirmed this with participants prior to testing by asking them to report what they saw when they viewed the stimulus monocularly. In the centre of each frame, a red fixation dot was presented. Fusion contours (width 0.8°) consisting of random noise pixels were also presented at the border of each frame to support binocular fusion of the two eyes’ images.

During the task, we presented face stimuli previously used in b-CFS experiments (Senju and Hasegawa 2005). These faces were monochrome digital photographs of four Asian adult females with neutral facial expressions. Eye gaze was either directed straight ahead (direct gaze) or away (averted gaze). All faces were equally adjusted in contrast and brightness and displayed in an oval (3.3° × 4.6°) to obscure hairlines. Edges of this aperture were blurred to assist suppression of the face stimuli during CFS masking.

### Procedure

Participants maintained fixation throughout the task. Each trial began with a 1 s presentation of the red frames, fusion contours, and fixation dots on a uniform black background. Following this, a suppressive multi-coloured Mondrian mask (updating at a frequency of 10 Hz) was introduced to the dominant eye, confirmed using the near convergence test (Rice et al. 2008), gradually ramping down linearly from 100% stimulus contrast to 0% stimulus contrast after 7 s. In the non-dominant eye, a face stimulus was gradually introduced by ramping up linearly from 0% to 100% stimulus contrast over a 1-s period. Participants were required to indicate whether the face stimulus was presented to the left or right of the central fixation point via arrow keys on the keyboard. No specific response about the gaze direction was required. In half of the trials, face stimuli were presented to the left of fixation (horizontal centre-to-centre distance 2.7°). In the other half of the trials, faces were presented to the right. Participants were instructed to respond as fast and as accurately as possible as soon as they located *any* part of the face. The time taken to make a response was used to indicate how long the stimulus took to break through the suppressive mask and reach conscious awareness (Tsuchiya and Koch 2005, Yang et al. 2007). Shorter suppression times were taken to indicate faster preconscious processing and prioritization of that stimulus by the visual system.

Participants completed 144 trials (72 direct gaze and 72 averted gaze) separated evenly into four blocks. Suppression times for each trial were recorded. Mean suppression times were calculated for direct and averted gaze stimuli based on correct trials only. Throughout the task, participants were seated 57 cm from the screen with their heads stabilized with a chin rest.

### Pre- and post-test questionnaires

Prior to being assigned to a group, participants completed a pre-test questionnaire to assess their state and trait anxiety (STAI; (Spielberger 1993)). Following the b-CFS task, participants also completed a post-test questionnaire, assessing any subjective change in state anxiety and situational self-awareness (SSAS; (Govern and Marsch 2001). In addition, participants were asked a set of questions to verify that those assigned to the “watched” group believed they were being watched during the task. Questions included, “Did you believe anyone was watching or monitoring you while you completed the experiment?” and “How strong was your feeling of being watched, surveilled, or monitored during the experiment?”. All participants were fully debriefed after testing.

### Control b-CFS experiment using neutral (non-face) stimuli

Based on findings from the main experiment, we included a subsequent control experiment to examine the specificity of our results towards faces. This control experiment was also designed to address the possibility that differences in demand characteristics induced by the presence of cameras would cue individuals in the watched group to respond quicker once aware of the stimulus, despite detecting it at a similar latency as individuals in the control group. To rule out the possibility that participants responded quicker to please the experimenter or some other social desirability bias, a new set of participants (*N* = 42) completed the original experiment but with *neutral* stimuli as opposed to face stimuli. The neutral stimuli were Gabor gratings oriented +15° or −15° (spatial frequency: 11.25 c/deg), the same size and mean contrast as the face stimuli. As with the main experiment, the edges of the stimulus aperture were blurred to assist CFS suppression. Participants completed 144 trials (72 oriented +15° and 72 oriented −15°) separated evenly into four blocks. We hypothesized that if demand characteristics induced by our experimental manipulation were underlying the results, we would observe the effects irrespective of a change in b-CFS stimulus.

### Analysis

Suppression times for the main experiment and the post hoc experiment were recorded as the time it took for participants to press a button to indicate the correct location of the stimulus. Data from trials where the participant responded incorrectly (i.e. indicated the wrong stimulus location) were excluded from the analyses. Statistical analyses were conducted using break suppression times in milliseconds using MATLAB 2023a (The MathWorks, Inc., Natick, Massachusetts, United States) and JASP (Love et al. 2019).

Data from participants failing to correctly localize the stimuli on more than 75% of trials were excluded from the analyses (i.e. *N* = 4 from each group in the main experiment and none in the post hoc experiment). Data from individuals scoring high on trait anxiety, i.e. above 39 (Julian 2011), were also excluded (i.e. *N* = 2 from each group in the main experiment), as high trait anxiety has previously been associated with quicker gaze processing under b-CFS conditions (e.g. Capitão et al. 2014, Chen et al. 2017).

## Results

### Main experiment

#### Participants believed they were being watched

Data from the post-test questionnaire verified that our experimental manipulation worked. Participants in the experimental group all answered yes to the question, “Were you aware of anyone watching, recording, or monitoring you while you completed the experiment?”. However, the majority (i.e. 24 of 26) only rated the feeling of being watched, surveilled, or monitored during the experiment as mild. Consistent with research demonstrating that humans have an innate bias to feel they are being observed despite the absence of a detectable observer (Titchener 1898, Freeman et al. 2005, Taylor et al. 2009, Bebbington et al. 2013, Burra et al. 2013, Mareschal et al. 2013, Harper and Timmons 2021), most participants in the control group (18 of 24) also reported at least a mild feeling of being watched during the experiment despite not being shown the surveillance cameras or being told they would be monitored. Interestingly, despite the conspicuous monitoring, all experimental participants reported they did not believe their task performance was affected.

#### Being watched led to faster perceptual processing of face stimuli

**Figure 2:**
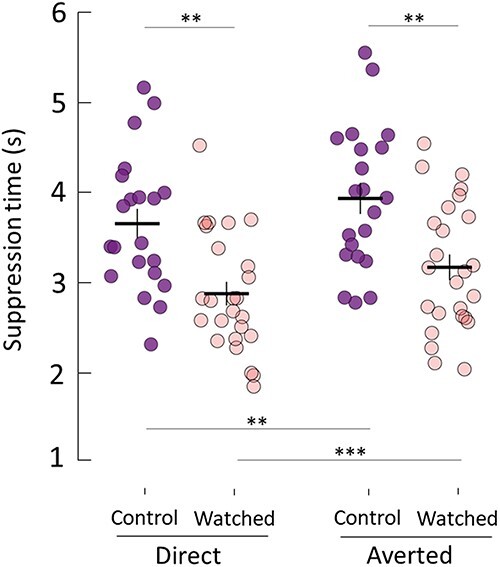
Being watched leads to faster awareness of faces. Participants in the watched group became aware of faces significantly faster than control participants. This was the case for both faces with direct and averted gaze. A replication of direct gaze advantage was also observed in each group. Horizontal lines denote group means. Vertical lines denote ±1 SEM. ** and *** represent p <0.01 and p < 0.001 respectively

We tested whether conspicuous surveillance influenced the speed at which faces with direct and averted gaze gained access to conscious awareness (Fig. 2). We found that participants in the “watched” group perceived faces significantly faster than control participants for both direct (*M* = 3.65 vs 2.87 s, respectively, *t*(44) = 3.74, *P* = 0.0011, CI = [0.29, 1.26], two-sample t-test Bonferroni adjusted, *d* = 1.10) and averted gaze direction (*M* = 3.93 vs 3.16 s, *t*(44) = 3.44, *P* = 0.0025, CI = [0.25, 1.28], two-sample t-test, Bonferroni adjusted, *d* = 1.01). We also found that faces with direct gaze were perceived significantly faster than faces with averted gaze, both for the control group (*M* = 3.65 vs 3.93s, *t*(20) = 3.79, *P* = 0.0023, CI = [0.1, 0.47], paired t-test, Bonferroni adjusted, *d* = 0.91) and the watched group (*M* = 2.87 vs 3.16 s, *t*(24) = 5.18, *P* = 0.0001, CI = [0.16, 0.43], paired t-test, Bonferroni adjusted, *d* = 0.94), which replicates previous findings of a direct gaze advantage (e.g. (Stein et al. 2011, Akechi et al. 2014, Seymour et al. 2016)).

#### No evidence of a speed-accuracy trade-off

Analysis of accuracy data revealed that participants’ accuracy in localizing face stimuli (either left or right from fixation, 50% chance) was high in both groups (*M* = 94.60%, SD = 6.2%), suggesting that the task instructions were followed. However, we found a difference in accuracy between groups, with the *watched* group being more accurate than the control group (two-sample t-test: *t*(44) = 2.78, *P* = 0.004, *d* = 0.827). This may provide additional evidence of enhanced stimulus processing from the “watched” group and rules out the possibility of a speed-accuracy trade-off.

#### Post-questionnaire ratings

With our watched group, we performed a paired t-test on pre- and post-state anxiety scores measured with the STAI (Spielberger 1993). The analysis revealed no significant difference *t*(25) = −0.303, *p* = 0.764, *d*  = −0.059, indicating that a *change* in anxiety induced by our experimental manipulation (i.e. a belief in being watched) was unlikely to explain our main results (Table 1). Differences in trait anxiety have also previously been shown to influence gaze processing under b-CFS (Capitão et al. 2014, Chen et al. 2017). To rule out the possibility that our watched group was generally more anxious than the control group, we conducted a two-sample t-test on Trait Anxiety scores and found no evidence for group differences, *t*(44) = −0.048, *P* = 0.962, *d* = 0.112 (Table 1).

**Table 1.** Anxiety and Self Awareness subjective reports.

	Control		Experimental	
	M	SD	M	SD
***STAI***				
Pre-State Anxiety	45.7	5.1	44.7	4.6
Post-State Anxiety	45.5	5.8	44.9	4.5
Trait Anxiety	47.9	5.3	47.9	3.3
***SSAS***				
Public	7.7	3.1	8.6	2.8
Private	10.0	2.7	11.1	2.3
Surroundings	12.4	1.9	11.9	2.0

A repeated measures ANOVA on self-awareness data (SSAS; (Govern and Marsch 2001)) also showed no statistically significant group differences in any of the three domains (i.e. Public, Surroundings, Private), *F*(2, 88) = 1.6, *P* = 0.208. This suggests that our finding of enhanced face processing in the watched group is unlikely due to a heightened sense of self-awareness (Baltazar et al. 2014, Conty et al. 2016), at least not at a level notable to our participants.

### Control experiment

#### No effects of being watched on the processing of neutral stimuli

**Figure 3:**
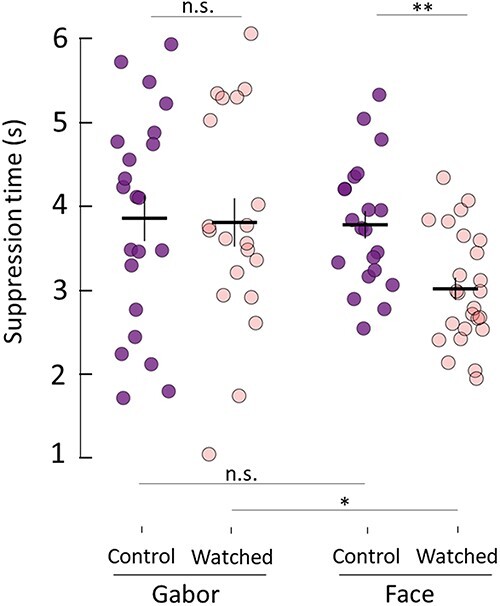
No effects of being watched on the processing of neutral stimuli. Being watched elicited no significant differences in suppression times associated with processing Gabor gratings compared to control participants. Results are averaged across orientations. For comparison, results from the main experiment are included (averaged across gaze direction) for each group. Horizontal lines denote group means. Vertical lines denote ±1 SEM. * and ** represent p < 0.05 and p <0.01 respectively. n.s. represents no significant difference

We conducted a post hoc control experiment to test the specificity of our results with regard to face processing and to address whether differences in demand characteristics may have biased individuals in the watched group to respond quicker once they became aware of the stimulus (Moors et al. 2019). Thus, in the post hoc experiment, we used neutral stimuli in the form of differently oriented Gabor gratings to test whether the effects of being watched on non-face stimuli were also evident. We hypothesized that such stimuli would be subject to the same social desirability bias (i.e. to perform the task faster and better when being watched) if this were indeed the basis of our main result. We found that running the same experiment with Gabor gratings did not elicit significant differences between the watched and control group (Fig. 3, *M* = 3.81 vs 3.86 s, *t*(40) = 0.12, *P* = 1, CI = [−0.88, 0.98], two-sample t-test on suppression times averaged across grating orientation, Bonferroni adjusted, *d* = 0.04). For comparison, suppression times for face stimuli (averaged across gaze directions) were significantly shorter for the watched group compared to controls (*M* = 3.02 vs 3.79 s, *t*(44) = 3.67, *P* = 0.0013, CI = [0.28, 1.26], two-sample t-test, Bonferroni corrected, *d* = 1.08). Comparing suppression times between neutral and face stimuli revealed that there were no significant differences for the control groups (*M* = 3.86 vs 3.79 s, *t*(41) = 0.21, *P* = 1, CI = [−0.69, 0.82], two-sample t-test, Bonferroni corrected, *d* = 0.06), but, again, the processing of faces elicited significantly shorter suppression times compared to neutral stimuli in the watched group (*M* = 3.81 vs 3.02 s, *t*(43) = 2.63, *P* = 0.02, CI = [0.09, 1.48], two-sample t-test, Bonferroni corrected, *d* = 0.76). The results of this control experiment provide evidence against a difference in demand characteristics across groups accounting for our original result, as such effects should be independent of stimulus type.

## Discussion

In this study, we examined the effects of surveillance on an essential function of human sensory perception—the ability to detect another person’s gaze. Previous research on ‘audience effects’ has shown that people tend to exhibit prosocial behaviours when they believe they are being watched (Hoffman et al. 1996, Haley and Fessler 2005, Bateson et al. 2006, 2015, Pfeiffer and Nowak 2006, Tourangeau and Yan 2007, Laidlaw et al. 2011, Risko and Kingstone 2011, Francey et al. 2012, Nettle et al. 2012, Powell et al. 2012, Baillon et al. 2013, Nasiopoulos et al. 2015, Oda et al. 2015, Conty et al. 2016, Bradley et al. 2018, Cañigueral and Hamilton de 2019). Here, we provide the first direct evidence that being watched also impacts involuntary perceptual processing of important sensory signals, namely facial cues to gaze direction. Specifically, we reveal that when people are conspicuously monitored via CCTV, they become aware of face stimuli much faster than when they are not monitored. This perceptual enhancement is almost a second faster in magnitude and seems to occur outside the individual’s awareness.

The ability to rapidly detect faces is of critical importance to human social interactions. Information conveyed in faces, such as gaze direction, enables us to construct models of other people’s minds and to use this information to predict behaviour (Baron-Cohen 1995, Emery 2000, Bayliss et al. 2011). Research suggests that humans have evolved dedicated neural mechanisms for detecting these important facial cues (Haith et al. 1977, Brothers 1990, Hood et al. 1998, Emery 2000, Farroni et al. 2002, Calder et al. 2007, Hietanen et al. 2008, Senju and Johnson 2009, Burra et al. 2013, Carlin and Calder 2013, Lawson 2015). Here, we show that the fundamental mechanisms underlying early face processing may be altered when one believes they are being watched. While previous research suggests a mere belief in being watched can strongly influence voluntary behaviour (Zhong et al. 2010, Risko and Kingstone 2011, Wykowska et al. 2014, Lawson 2015, Nasiopoulos et al. 2015, Wang and Apperly 2017, Cañigueral and Hamilton de 2019, Colombatto et al. 2019), our data reveal this may also impact the more fundamental involuntary aspects of human perception and action.

Our finding that sensory processing of gaze direction is facilitated by the act of being watched is consistent with evidence suggesting top-down cognition can influence the earliest stages of gaze processing (Teufel et al. 2009). Also, eye-tracking studies indicate that a social presence can significantly alter where attention is allocated (Risko and Kingstone 2011, Nasiopoulos et al. 2015). In light of our findings, an enhanced and specific allocation of attentional resources towards self-relevant social information seems plausible. Importantly, our results rule out that being watched leads to a non-specific attentional boost, as non-face stimuli did not benefit from this effect; instead, our results support the idea that this is a specific effect directed towards face information. This is consistent with clinical observations of social-specific attentional biases and a hyper-sensitivity to eye gaze in mental health conditions like psychosis and social anxiety disorder where individuals hold irrational beliefs or preoccupations with the idea of being watched (Rosse et al. 1994, Hooker and Park 2005, Corlett et al. 2009, Tso et al. 2012, Langer and Rodebaugh 2013, Chen et al. 2017, Langdon et al. 2017, Stuke et al. 2021). Future investigations should explore in detail the effects of surveillance and the sense of privacy on public mental health, as these can have profound social implications (Aboujaoude 2019).

In relation to attention, recent research, both in humans and other social animals, has highlighted that being watched has opposite effects on performance depending on the task’s difficulty. The presence of peers has an impact on attention resources, improving performance in simple or well-learned tasks while disrupting complex or poorly learned tasks (for a review, see (Belletier et al. 2019)). Neuroimaging results support this dichotomy, indicating that the presence of peers leads to changes in domain-general regions but not in task-specific areas (Tricoche et al. 2023). These observations are consistent with our results, as our task relies on an automatic ability to detect eye gaze, an ability that has evolved to be as simple and robust as possible due to its important ecological value. Therefore, according to the effects of being watched on attention mentioned above, gaze detection is expected to be improved by the belief in the presence of watching peers, as our results show. Future research should delve deeper into the impacts of surveillance on perception and attention as well as its effects on performance.

It is important to note that higher-level audience effects and demand characteristics commonly reported in the literature cannot explain the current results. Since our study probed early and involuntary aspects of face processing (Tsuchiya and Koch 2005, Yang et al. 2007, Stein et al. 2011), it is unlikely that participants could simply control their response times to conform to experimenter expectations. More importantly, our post hoc control experiment using neutral stimuli revealed no differences in stimulus processing times between the watched and control groups. If demand characteristics or changes in anxiety or arousal levels induced by our experimental manipulation were to explain our results, one would expect to see such factors also impacting the processing of other stimuli, not just faces.

Over the past decade, technological advancements have resulted in increasingly powerful forms of human surveillance. We now live in a society with CCTV and face recognition technology, trackable mobile devices, and the monitoring of our activities through artificial intelligence and the ‘Internet of Things’. Emerging technologies, like commercially available neurotechnology, will present new forms of mental surveillance. The data we present in this study suggest that surveillance has a direct impact on an essential human faculty—the ability to rapidly detect eye gaze signals. Given the significance of this capacity for human social interaction, these results highlight important implications for public mental health. What is also interesting to note is that participants reported the feeling of being watched as only very mild despite there being a number of cameras directed at them during the experiment. This is consistent with findings from recent surveillance research showing that people quickly become normalized to being monitored (Oulasvirta et al. 2012, Nasiopoulos et al. 2015). Additionally, a recent meta-analysis has pointed out that the presence of ‘watching eyes’ or eye-like stimuli is more than twice as successful at preventing antisocial behaviour compared to the presence of cameras, with a 35 vs 16% reduction of antisocial behaviour, respectively (Dear et al. 2019). This highlights the strong influence of gaze on human behaviour and insists on the need for a better understanding of the interaction of gaze perception and surveillance in both conscious and unconscious behaviour.

Here, we provide a surprising yet unsettling finding that despite the report of little concern or preoccupation with being monitored, its effects on basic social processing are marked, highly significant, and imperceptible to the participants.

## Data Availability

Data and MATLAB scripts to reproduce experimental figures and statistics are available in OSF at https://dx.doi.org/10.17605/OSF.IO/Q3GHJ.
